# Characterization of the complete chloroplast genome and phylogenetic analysis of *Silene jenisseensis* (Caryophyllaceae)

**DOI:** 10.1080/23802359.2019.1704654

**Published:** 2020-01-10

**Authors:** Li-Zhen Ling

**Affiliations:** School of Biological Sciences and Technology, Liupanshui Normal University, Liupanshui, China

**Keywords:** Caryophyllaceae, chloroplast genome, phylogenetic analysis, *Silene jenisseensis*

## Abstract

The first complete chloroplast genome (cp) sequences of *Silene jenisseensis* were reported in this study. The *S. jenisseensis* cp genome was 150,299 bp in size, with two inverted repeat (IR) regions of 25,384 bp, the large single-copy (LSC) region of 82,153 bp, and the small single-copy (SSC) region of 17,378 bp. The cp genome of this species contained 111 genes, including 77 protein-coding genes, 4 ribosomal RNA, and 30 transfer RNA genes. The overall GC content was 36.4%. Phylogenetic analysis of the cp genomes within the Caryophyllaceae family suggests that *S. jenisseensis* is closer to the clade formed by *S. capitata* and *S. aprica*.

*Silene jenisseensis* Willdenow is perennials of the Caryophyllaceae family and broadly distributed in forest and meadow communities of Russian, Mongolia, China, and Korea (Zhou et al. 2001). The root of this species is well known in traditional Chinese medicine as a substitute for the Chinese drug Yin-Chai-Hu (root of *Stellaria dichotoma* Linnaeus var. *lanceolata* Bge) and it is utilized to treat fever due to Yin-deficiency and fever in infant malnutrition (Chinese Pharmacopoeia 1990). Phytochemical studies have reported that ecdysteroids, flavonoids, triterpene glycosides, and sterols are the major characterized constituents of this plant (Darmograi 1977; Cui and Qiao 1995; Lacaille-Dubois et al. 1995; Olennikov and Kashchenko 2017). Here, we characterized the complete chloroplast (cp) genome of *S. jenisseensis* based on the Illumina sequencing technology to understand its genetic background and to explore its phylogenetic placement.

The specimen (CaoW5049) of *S. jenisseensis* was collected from Fengcheng, Liaoning, China (N40°22′52″, E124°5′1″, 110 m) and deposited in the herbarium of Kunming Institute of Botany, CAS (KUN). The total DNA was extracted and used for sequencing as previously described (Zhang et al. 2019). The 2 Gb raw data were generated and used for *de novo* cp genome assembly with SPAdes (Bankevich et al. 2012) and all predicted genes were annotated using PGA (Qu et al. 2019). The complete cp genome sequence of *S. jenisseensis* was deposited in GenBank database under the accession number MN723869.

The complete cp genome of *S. jenisseensis* is 150,299 bp in length and shows the GC content of 36.4%. The cp genome of this species displays a typical quadripartite structure, two copies of inverted repeats (IRs, 25,384 bp each) segregated by a large single-copy (LSC, 82,153 bp) region and a small single-copy (SSC, 17,378 bp) region. In addition, a total of 111 unique genes were encoded, including 77 protein-coding genes (PCGs), 30 transfer RNA (tRNA) genes, and 4 ribosomal RNA (rRNA) genes. Of them, five PCGs (*ndhB*, *rpl2*, *rps12*, *rps7*, and *ycf2*), four rRNAs (*rrn16*, *rrn23*, *rrn4.5*, and *rrn5*), and seven tRNAs (*trnA-UGC*, *trnI-CAU*, *trnI-GAU*, *trnL-CAA*, *trnN-GUU*, *trnR-ACG*, and *trnV-GAC*) have two copies. Fourteen genes (*atpF*, *ndhA*, *ndhB*, *petB*, *petD*, *rpl16*, *rpoC1*, *rps16*, *trnA-UGC*, *trnG-UCC*, *trnI-GAU*, *trnK-UUU*, *trnL-UAA*, and *trnV-UAC*) contain one intron and three genes (*clpP*, *rps12*, and *ycf3*) have two introns.

To determine the phylogenetic position of *S. jenisseensis*, a phylogenetic analysis was carried out with the maximum likelihood (ML) method (Stamatakis 2014). *Agrostemma githago* from the Caryophyllaceae family was used as outgroup. The cp genomes of *S. jenisseensis* and previously published other species from the genus *Silene* were used for phylogenetic analysis in this study. The GenBank accession numbers of all the species used were provided in Figure 1. The phylogenetic tree showed that *S. jenisseensis* is more closely related to the clade formed by *S. aprica* and *S. capitate* (Figure 1). The *S. jenisseensis* cp genome reported in this study may provide useful resources for the development of medical value as well as robust phylogenetic study at deep level of *Silene* in the future.

**Figure 1.**
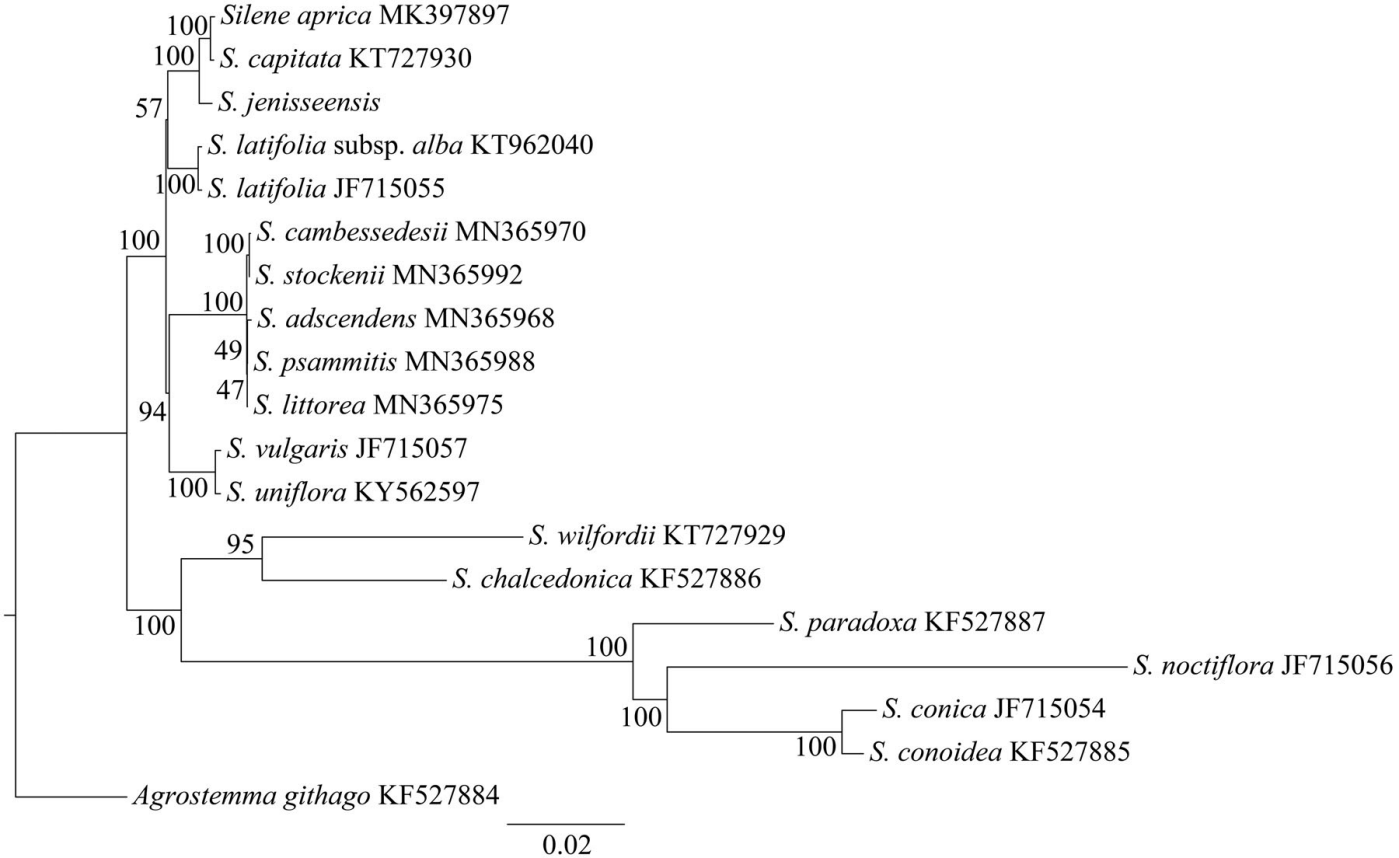
The maximum likelihood (ML) tree of *Silene* inferred from the complete chloroplast genome sequences. Numbers at nodes correspond to ML bootstrap percentages (1000 replicates).
